# Interpretability and performance of a 3D C-vit model for accurate grading of pediatric brain tumors

**DOI:** 10.3389/fonc.2026.1763280

**Published:** 2026-04-27

**Authors:** Huixin Wu, Limeng Zhao, Yong Zhang, Can Zhang, Guohua Zhao, Wenjing Li, Yangyang Cheng, Xinxin Wang, Tan Ping, Xinyu Wang, Fupeng Wei, Qian Zhang, Jie Dong, Weijian Wang

**Affiliations:** 1 School of Information Engineering, North China University of Water Resources and Electric Power, Zhengzhou, China; 2 Institute of Aerospace Information, Henan Academy of Sciences, Zhengzhou, China; 3 Department of Magnetic Resonance Imaging, The First Affiliated Hospital of Zhengzhou University, Zhengzhou, China; 4 School of Artificial Intelligence, Zhongyuan University of Technology, Zhengzhou, China

**Keywords:** feature interpretability, grading, MRI, pediatric brain tumor, transformer

## Abstract

**Background:**

Accurate preoperative grading of pediatric brain tumors is crucial for formulating individualized treatment plans. Traditional methods rely on subjective experience, while existing deep learning models have limitations in capturing long-distance dependencies and local details. This study aims to develop and validate an innovative 3D hybrid deep learning model (3D C-Vit) for pediatric brain tumor grading and analyze its performance and interpretability.

**Methods:**

This retrospective study included 340 cases of pediatric brain tumors (143 low-grade cases and 197 high-grade cases). Tumor regions were independently annotated by two senior radiologists with consistency achieved. The data were divided into training, validation, and test sets in a ratio of 70:15:15. The model input included five MRI sequences: CE-T1WI, T1WI, T2WI, FLAIR, and ADC. The proposed 3D C-Vit model integrates the Channel Attention-Enhanced Feature Fusion (CAEFF) module, Multi-Scale Feature Extraction (MSFE) module, and Multi-Head Self-Attention (MHSA) mechanism. Model performance was evaluated using AUC, accuracy (ACC), precision, recall, and F1-score. Chi-square test and LASSO regression were used for feature selection and interpretability analysis.

**Results:**

The 3D C-Vit model performed optimally on the test set: AUC was 91.36%, ACC was 86.53%, and F1-score was 89.29. Ablation experiments confirmed that CAEFF, MSFE, and MHSA modules increased ACC by 6.92%, 11.67%, and 1.64%, respectively, and AUC by 6.79%, 11.14%, and 1.66%, respectively. Among the radiomics models, LASSO regression screened out 59 key features. The 3D C-Vit model was significantly superior to the clinical model (ACC 69.23%, AUC 79.09%) and the best radiomics models (SVM, ACC 77.55%, AUC 86.14%) in all assessment metrics.

**Conclusion:**

The 3D C-Vit model proposed in this study can effectively and automatically grade pediatric brain tumor, and its performance significantly surpasses traditional clinical methods and existing radiomics models. The model combines the local feature extraction capability of CNN with the global modeling advantage of Transformer and effectively improves the grading accuracy through the innovative CAEFF and MSFE modules. Its high accuracy and interpretability provide clinicians with a reliable preoperative tumor grading tool, which is helpful for quickly formulating precise individualized treatment plans.

## 1 Introduction

Brain tumor is one of the most common solid tumor in children, and its accurate grading is crucial for formulating individualized treatment strategies and evaluating prognosis (1). According to the classification of the World Health Organization (WHO), brain tumor is divided into four grades. Low-grade (grade I/II) can be cured by surgery, while high-grade (grade III/IV) tumor have a high degree of malignancy, fast growth rate, and are prone to relapse, often requiring treatments such as surgery, radiotherapy, and chemotherapy (2). Therefore, accurate preoperative differentiation of tumor grades is of decisive significance for optimizing treatment decisions and improving patients’ quality of life. Magnetic resonance imaging (MRI), with its high resolution and multimodal imaging capabilities, plays an important role in the detection, localization, and evaluation of morphological characteristics of brain tumor (3).However, traditional diagnostic methods rely heavily on physician experience and suffer from high subjectivity and low efficiency, making it difficult to meet clinical needs. In recent years, deep learning technology has demonstrated significant potential in medical image analysis, capable of improving the automation and accuracy of tumor grading; however, existing deep learning models still face numerous challenges when processing the complex features of pediatric brain tumors (4). Therefore, developing a non-invasive technology that can efficiently and automatically identify tumor grades with good interpretability is of great significance for improving the preoperative diagnostic efficacy and personalized treatment planning ability of pediatric brain tumor.

Convolutional Neural Networks (CNNs), with their powerful local feature extraction capability, have become the mainstream tool for medical image analysis and have performed excellently in brain tumor grading tasks (5–7). Liu et al. (8) proposed a multimodal deep learning approach that integrates multimodal information via a cross-attention mechanism, substantially improving diagnostic performance for brain cancer. Ahmad et al. (9) developed a CNN-based classifier that leverages transfer learning and fine-tuning techniques to achieve a high detection accuracy on MRI images. Researchers have effectively mitigated the vanishing gradient problem in deep network training—and enhanced model focus on critical lesion regions—by refining network architectures and incorporating attention mechanisms. Although CNNs excel at capturing local textures, edges, and spatial structures, their inherent local receptive field limits their capacity to model long-range dependencies within tumors and to accommodate inter-patient variations in texture, shape, and size (10–12).This limitation is particularly pronounced in pediatric brain tumors, which exhibit highly variable morphologies, complex internal structures, and are frequently accompanied by peritumoral edema (13).

To overcome the limitations of CNNs in modeling global semantics, the Transformer architecture, based on self-attention mechanisms, has demonstrated unique advantages in the field of medical image analysis (14). The Transformer can model correlations between arbitrary positions within an image, aiding in the understanding of a tumor’s extent of invasion, its spatial relationship with functional regions, and its overall pathological characteristics. However, the standard Transformer may result in the loss of local details when processing high-resolution images, and its high computational complexity limits its performance in capturing minute infiltrative foci and boundary details in pediatric tumors (15). Consequently, hybrid architectures that combine the local perception capabilities of CNNs with the global dependency modeling advantages of Transformers have become a key research direction in brain tumor grading. Linh T. Duong et al. (16) were the first to integrate EfficientNet with Transformers to enable multimodal medical image analysis, providing a paradigm for subsequent research (10, 17–19). Exploring methods to integrate Transformer components into various CNN architectures. However, existing hybrid models still face critical challenges in pediatric brain tumor tasks, namely how to efficiently integrate information from multi-parameter MRI sequences and how to enhance sensitivity to the tumor’s internal multiscale structures while maintaining global dependency modeling (20).

In the field of medical artificial intelligence, model interpretability is a key factor determining its potential for clinical translation (21, 22). For the high-risk task of pediatric brain tumor grading, models must possess transparent and trustworthy decision-making processes so that clinicians can understand the basis for judgments and adjust treatment plans accordingly. However, most existing deep learning methods operate as “black boxes” and lack intuitive explanations, which not only hinders clinical application but also limits algorithm optimization.

To address these issues, this study proposes a 3D hybrid deep learning model—3D C-Vit—for the precise automatic grading of pediatric brain tumors. The model’s innovations include: (1) CAEFF module: introducing a channel attention mechanism (SE) to enhance feature fusion across multi-parameter MRI sequences (CE-T1WI, T1WI, T2WI, FLAIR, ADC); (2) MSFE module: Utilizes a gated network and deep separable group convolution (DSGC) to achieve cross-scale dynamic feature extraction, improving sensitivity to intratumoral multiscale structures and computational efficiency; (3) 3D hybrid architecture: Combines the local 3D feature extraction capability of CNNs with the global 3D context modeling capability of Transformers (multi-head self-attention, MHSA), with feature fusion performed via a feedforward neural network (MLP).

Furthermore, this study conducted interpretability analyses on the model and radiomic features, revealing decision mechanisms through attention visualization and radiomic comparisons. In summary, the main contributions of this study are as follows:

1. We propose the CAEFF and MSFE modules, which effectively enhance multi-sequence feature fusion and cross-scale feature extraction capabilities while reducing computational complexity;
2. We construct the 3D C-Vit hybrid architecture, achieving a balance between local perception and global modeling, and providing a new approach for the precise grading of pediatric brain tumors;
3. We conduct in-depth interpretability analysis to enhance clinical confidence in the model and provide guidance for clinical decision-making.

## 2 Materials and methods

### 2.1 Introduction to data sets

**Figure 1:**
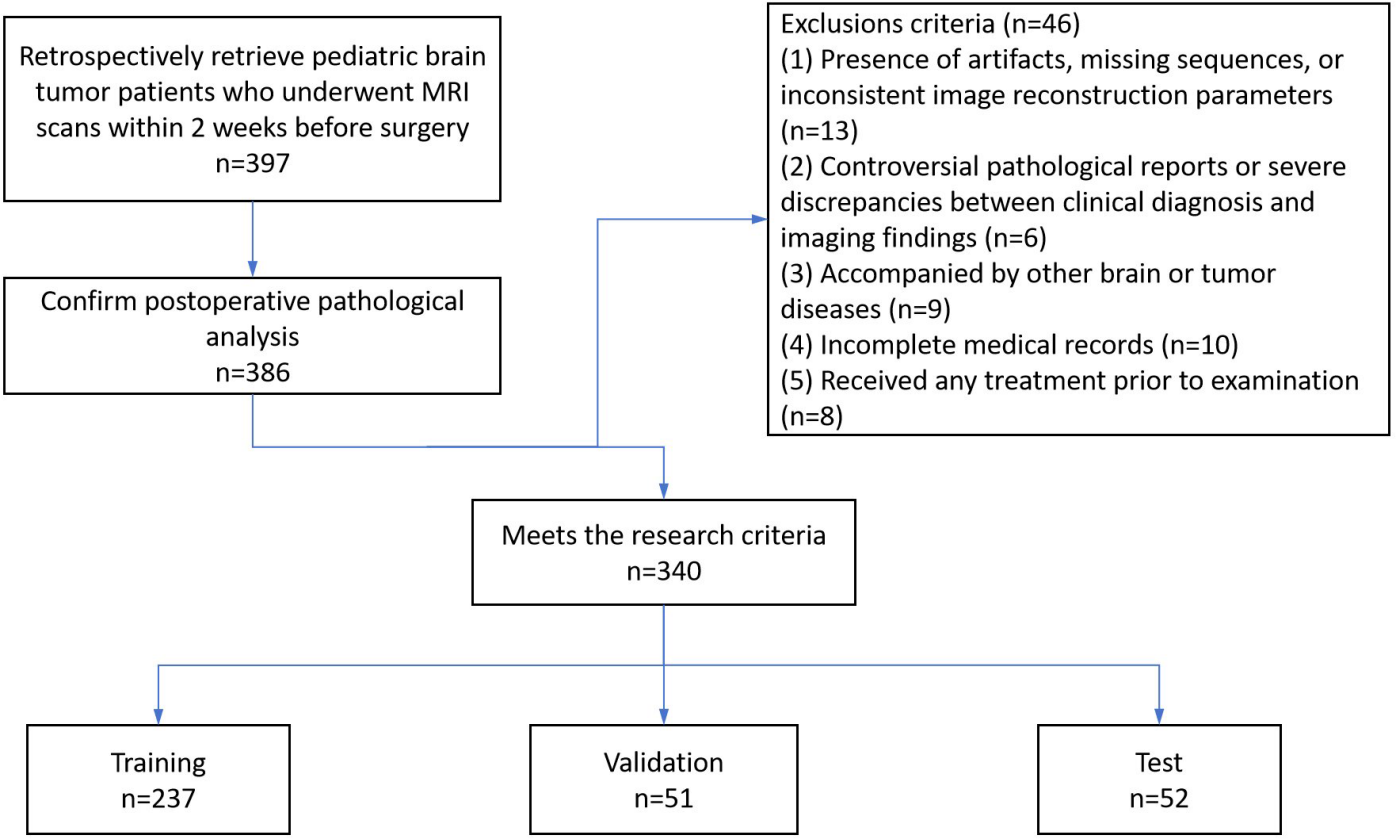
Patient collection flow chart.

This study is a retrospective study, and the data were collected from the First Affiliated Hospital of Zhengzhou University. The study protocol was approved by the institutional ethics committee, and informed consent was waived. The inclusion criteria included: (1) pediatric patients (age ≤ 18 years) with brain tumor confirmed by postoperative histopathology; (2) complete preoperative multi-parametric MRI images (CE-T1WI, T1WI, T2WI, FLAIR, ADC). The exclusion criteria included: (1) presence of artifacts, missing sequences, or inconsistent image reconstruction parameters; (2) controversial pathological reports or serious inconsistency between clinical diagnosis and imaging findings; (3) accompanied by other brain or tumor diseases. As shown in **Figure 1**, a total of 340 children with pathologically confirmed brain tumor were finally included, among whom 143 cases were low-grade (WHO grade I/II) and 197 cases were high-grade (WHO grade III/IV).Patients were randomly divided into a training set (n=237), a validation set (n=51), and a test set (n=52) in a 70:15:15 ratio. The distribution of patients’ baseline characteristics (such as age, gender, tumor location, type) was balanced among the three cohorts (see **Table 1** for details).

**Table 1:** Patient characteristics.

Characteristics	Low_grade	High_grade	*P* value
N, n (%)	142 (41.8)	198 (58.2)	
Gender, n (%)			0.504
Male	76 (53.5)	130 (65.7)	
Female	66 (46.5)	68 (34.3)	
Mean age[92%CI], years	6.5 [1.2-16]	8.3 [0.6-15.4]	0.734
Location, n (%)			<0.001
The fourth ventricle	56 (39.4)	144 (72.8)	
Vermis/Midline	15 (10.6)	23 (11.6)	
Brainstem	6 (4.2)	4 (2.0)	
Cerebellum Hemisphere	64 (45.1)	22 (11.1)	
Occipital Lobe	0 (0.0)	4 (2.0)	
Frontal Lobe	1 (0.7)	1 (0.5)	
Type, n (%)			<0.001
EP	33 (23.2)	39 (19.7)	
MB	0 (0.0)	159 (80.3)	
PA	109 (76.8)	0 (0.0)	
Necrosis, n (%)			<0.001
Necrotic	30 (21.1)	159 (80.3)	
Non-necrotic	112 (78.9)	39 (19.7)	
Edema, n (%)			<0.001
Edematous	62 (43.7)	135 (68.2)	
Non-edematous	80 (56.3)	63 (31.8)	
Ki-67, n (%)			<0.001
>15%	4 (2.8)	198 (100)	
≤15%	138 (97.2)	0 (0.0)	

EP, Ependymoma; MB, Medulloblastoma; PA, Pilocytic Astrocytoma.

**Figure 2:**
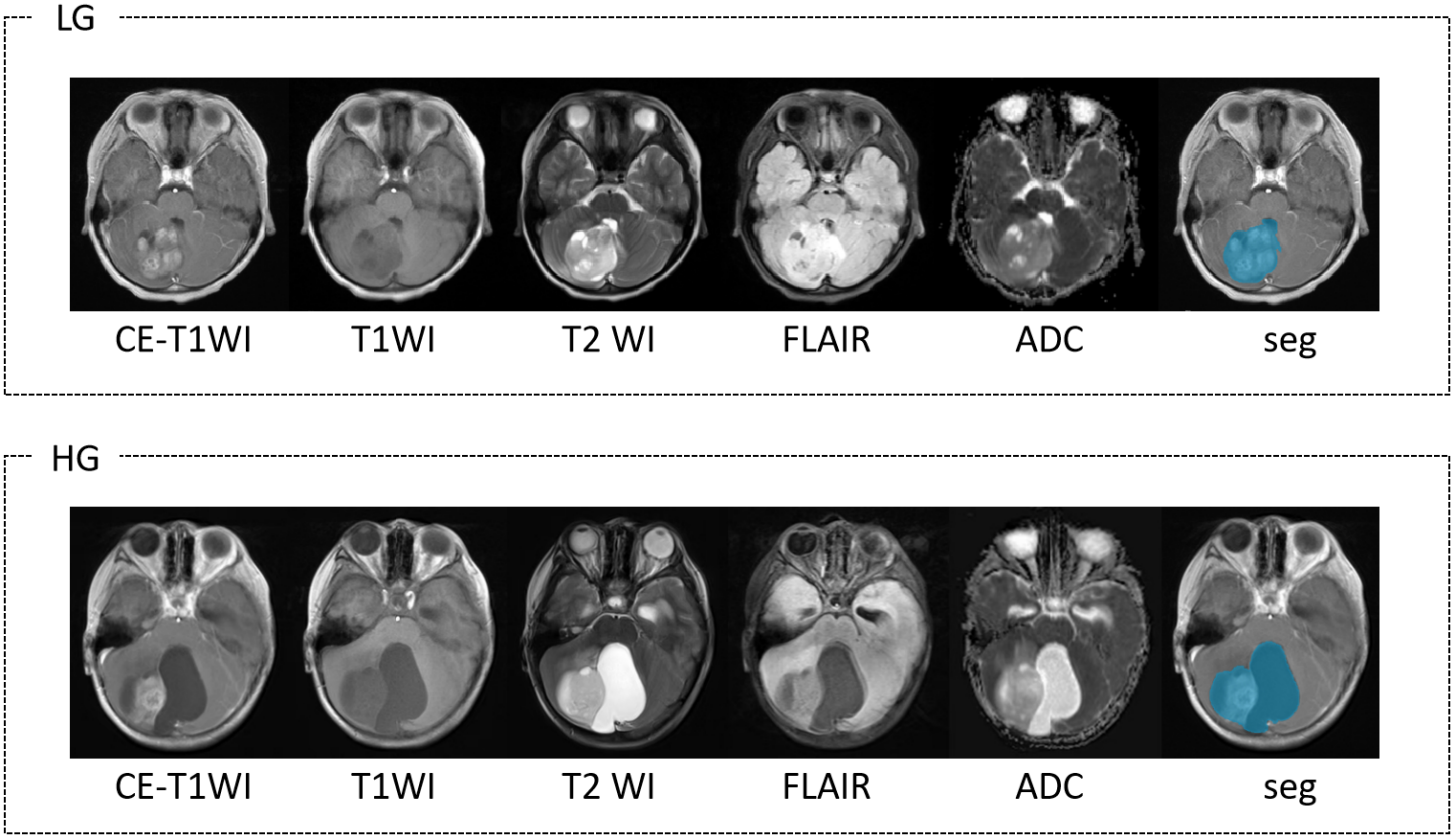
Multi-sequence MRI image comparison of low-grade (LG) and high-grade (HG) brain tumor. Show the CE-T1WI, T1WI, T2WI, FLAIR, ADC sequences and tumor segmentation (Seg) results in sequence, and the blue area represents the tumor area.

As shown in **Figure 2**, the dataset includes five sequences scanned by MRI: contrast-enhanced T1-weighted imaging (CE-T1WI), T1-weighted imaging (T1WI), T2-weighted imaging (T2WI), fluid-attenuated inversion recovery sequence (FLAIR), and apparent diffusion coefficient (ADC). All preoperative MRI images were acquired from 3.0-T scanners. The detailed scanning agreement is shown in the **Supplementary Material**.

### 2.2 Data preprocessing

Bias correction was performed using N4 bias correction. MRI images were resampled with a voxel size of 0.5×0.5×5 mm³, and T1WI, T2WI, FLAIR, and ADC images were registered to the CE-T1WI sequence. Image intensity normalization was performed using the WhiteStripe method in the CaPTK software (https://cbica.github.io/CaPTk/). This method identifies white matter tracts in the image, calculates linear transformation parameters based on the intensity distribution in these regions, and then normalizes the entire image. This process eliminates intensity variations between different subjects and scanning conditions, ensuring the comparability of image intensities and providing a consistent standard for subsequent analysis. Our model training is based on tumor regions manually annotated by physicians. Two radiologists with over five years of experience independently delineated the tumor regions on T2WI images using 3D Slicer software (https://www.slicer.org/) and reached consensus. In cases of disagreement or discrepancy, a third radiologist was consulted to provide additional input and help reach a consensus.

### 2.3 Model construction of 3D C-Vit

**Figure 3:**
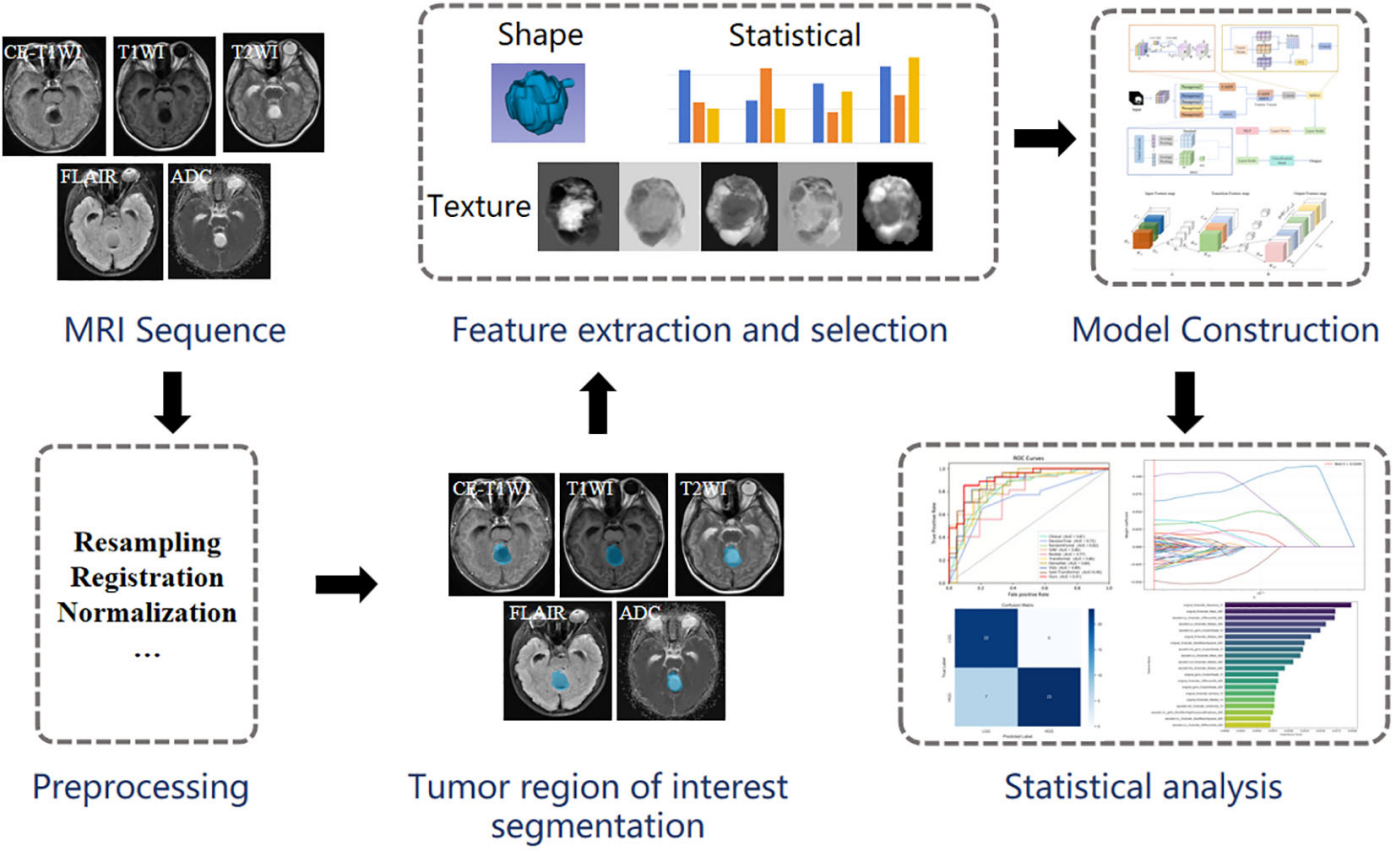
Overall process diagram. First, resample, register and normalize the pre-process of the original image, outline the region of interest and segment it, then extract and select the tumor region features, construct the deep learning model, and finally conduct statistical analytics.

This study proposes and constructs a novel hybrid 3D neural network architecture based on the collaborative optimization of Transformers and CNN, with the aim of effectively extracting local features and understanding contextual dependencies for accurate classification. The proposed method is illustrated in **Figure 3**.

**Figure 4:**
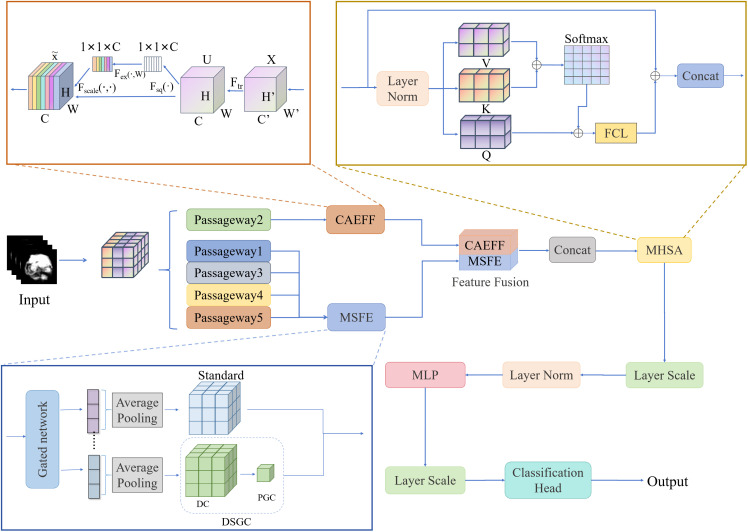
3D C-Vit model architecture.

The overall architecture of the 3D C-Vit model is shown in **Figure 4**. Its core innovations lie in three modules: Channel Attention Enhancement Feature Fusion (CAEFF), Multi-Scale Feature Extraction (MSFE), and Multi-Head Self-Attention (MHSA) mechanism. The model input is the pre-processed five-channel 3D MRI volume (corresponding to CE-T1WI, T1WI, T2WI, FLAIR, ADC sequences respectively).To reduce the incompleteness of local feature extraction by convolution, the second channel enters the Squeeze-and-Excitation (SE) attention to extract features. The remaining channels are controlled by a gating network, where channels containing important tumor features use standard convolution, and channels with non-important tumor features are passed to depthwise separable grouped convolution. This mock-up takes advantage of the Multi-Head Self-Attention (MHSA) mechanism to extract global features. The extracted features are passed to the Multi-Layer Perceptron (MLP) to Fusion global and local features, and then output the outcome after two layer scaling and classification head, with layer normalization operation executed after the first layer scaling.

#### 2.3.1 Image input layer and model depth

The 3D C-Vit model begins with an image layer containing model inputs. The image input layer is responsible for receiving preprocessed multimodal brain MRI image data. This layer is designed with five input channels, each corresponding to an MRI sequence, in order: CE-T1WI, T1WI, T2WI, FLAIR, and ADC.

The model consists primarily of multiple layers. Each block (as shown in **Figure 4**, from the MHSA to the MLP section) constitutes one layer of the Transformer encoder, with a total of 8 layers. The overall architecture of the model is formed by stacking multiple blocks. Given that the model has a channel dimension of 768 and 12 attention heads, the attention head size is 64.

#### 2.3.2 CAEFF

**Figure 5:**
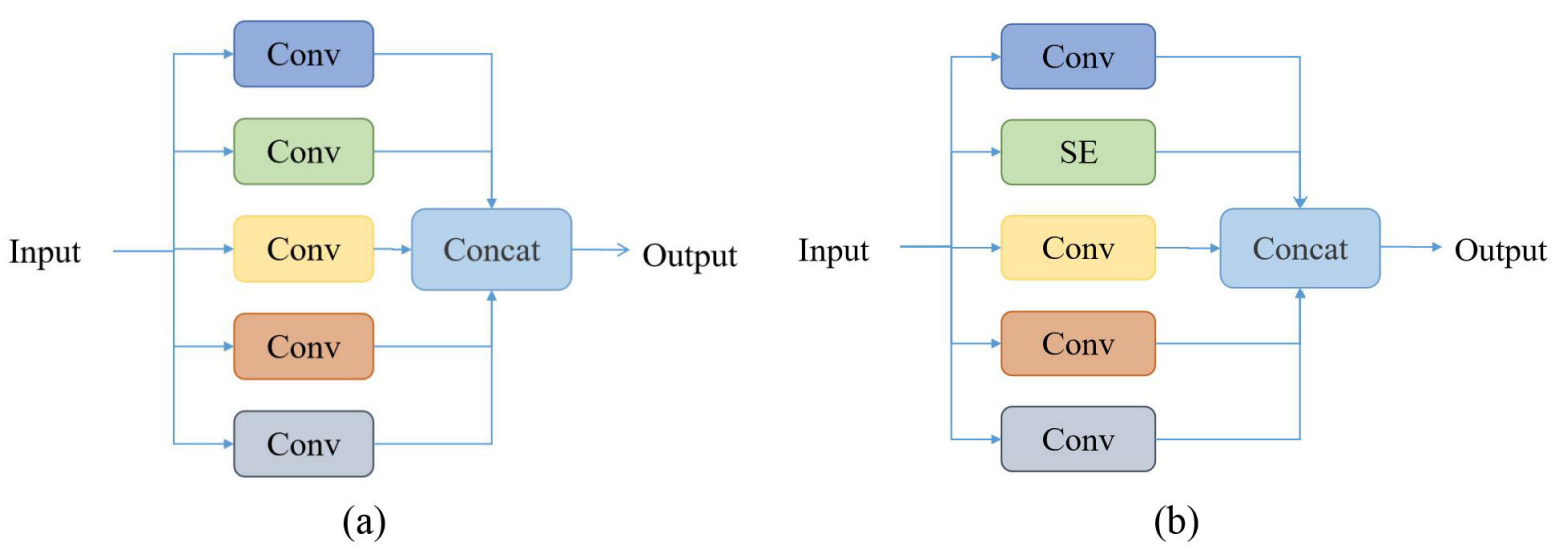
CAEFF block. **(a)** The original channel inputs five standard convolutions. **(b)** the second channel is replaced by SE attention.

**Figure 6:**
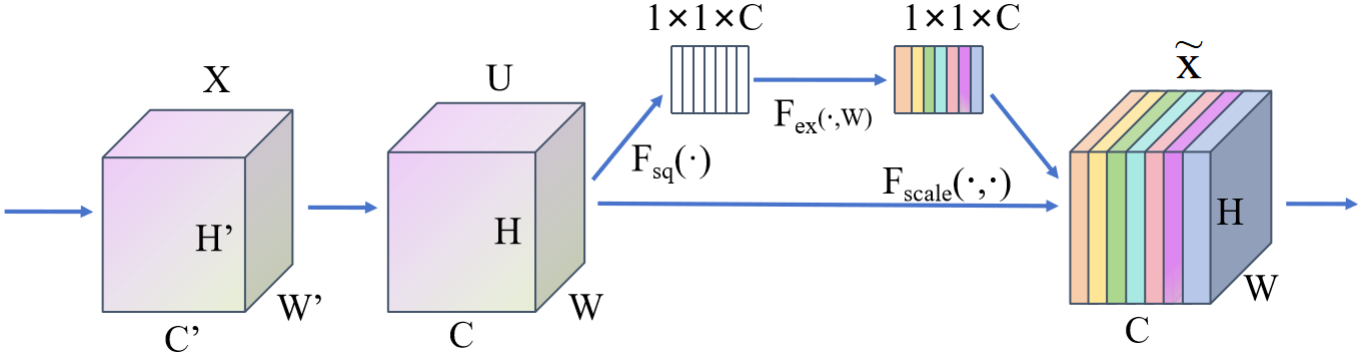
SE diagram of attention mechanism.

CAEFF block is shown in **Figure 5**, the SE attention mechanism can not only focus on critical modalities and subtle pathological signals through channel-level weight learning but also adapt to the requirements of limited data volume and 3D modeling with extremely low computational cost. By integrating SE attention, the T1WI sequence addresses the issues of spatial misalignment and artifact interference that sequences like Flair and T1c encounter in tumor localization, while simultaneously providing a stable anatomical reference for multi-sequence feature fusion. Therefore, the second module within the multi-path convolution block is replaced with the SE attention mechanism, which then receives the T1WI sequence as input. As shown in **Figure 6**, this mechanism compresses the spatial information of the feature map through global average pooling (Squeeze) to generate a global description at the channel level. Then, a simple fully connected network (Excitation) calculates the importance weights for each channel, and finally, these weights are applied to the original feature map. Here, X∈R^(H"×C"×W") represents the input feature map, U∈R^(H×C×W) represents the output feature map, and ${\text{F}}_{\text{tr}}$ is treated as a standard convolution operator, denoting the process of generating the feature map from X to U. ${\text{F}}_{\text{sq}}$ refers to full-channel average pooling, ${\text{F}}_{\text{ex}}$ denotes the weight calculation process, and ${\text{F}}_{\text{scale}}$ denotes the reweighting operation, ultimately producing the output feature map $\tilde{\text{X}}$.

#### 2.3.3 MSFE

Convolutional, pooling, input, and fully connected layers make up CNN, a deep learning model. As the central component, the convolutional layer exhibits strong representation capabilities when processing structured image features and performs joint spatial-channel modeling of input feature maps using shared convolutional kernels. Dense connections are used in conventional convolutional layers. Although this makes it possible to build deep networks by stacking tens to hundreds of convolutional layers to improve feature abstraction capabilities, as the number of layers rises, this fully connected architecture encounters two significant difficulties. On the one hand, the parameter size increases quadratically, which raises the computational and storage expenses significantly. However, the chain rule may cause gradients to accumulate numerical instability during backpropagation.

**Figure 7:**
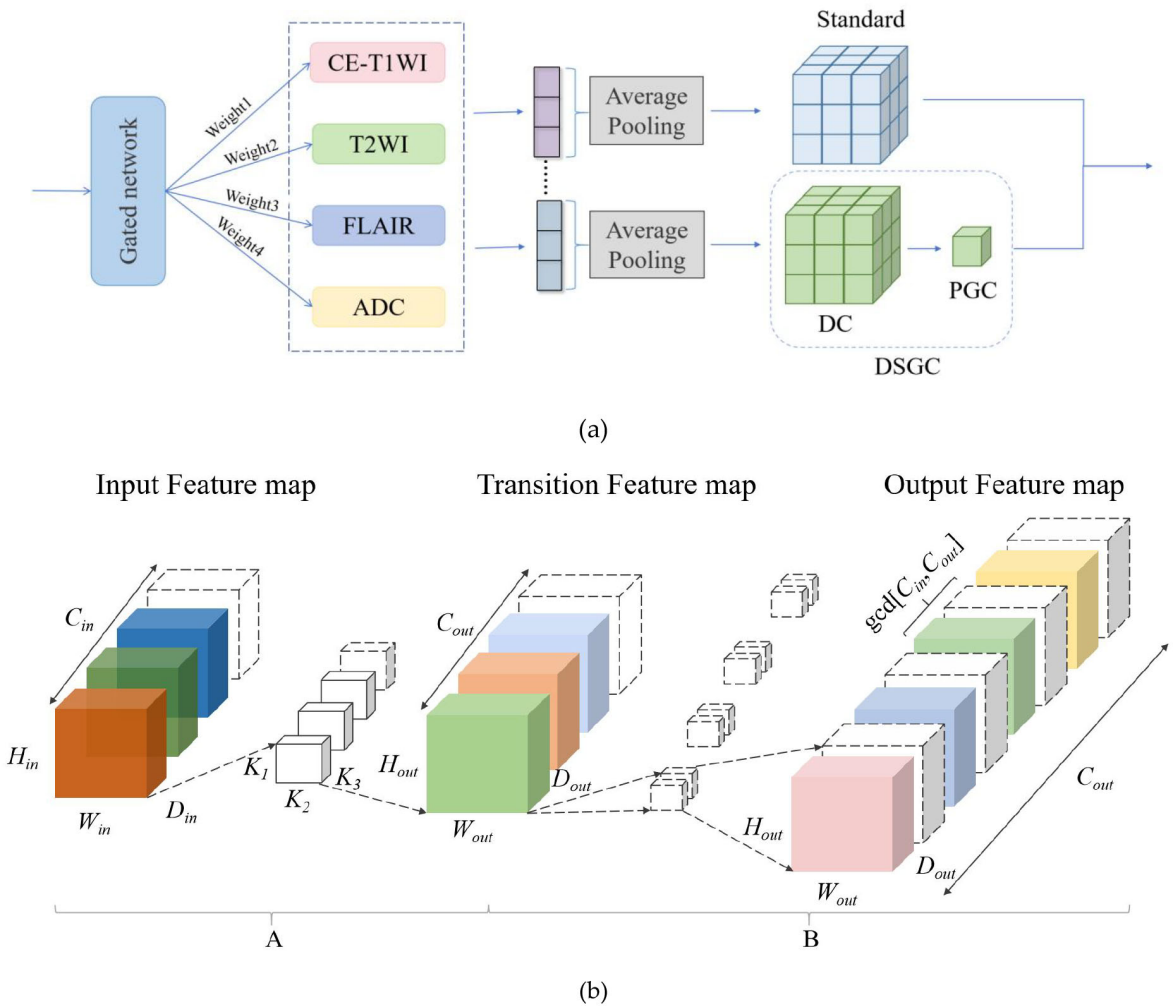
**(a)** MSFE working principle diagram. **(b)** DSGC working principle diagram. A, Deep convolution (DC); B, Point-by-point grouped convolution (PGC).

To overcome the efficiency bottleneck of traditional convolutions and enhance multi-scale feature expression capabilities, we propose the Multi-Scale Feature Extraction (MSFE) module, whose structure is shown in **Figure 7a**. This architecture introduces a gate network (GN) before the DSGC module to achieve cross-scale information fusion through dynamic feature modulation. The MSFE comprises two core modules:

1. GN: A lightweight attention mechanism is employed to capture the global context of input feature maps through global average pooling, thereby generating spatial-channel joint attention weights. These weights calibrate the input feature maps via element-wise multiplication, enabling dynamic enhancement or suppression of feature responses to differentiate the importance levels for tumor grading.
2. Deep Separable Group Convolution (DSGC) (23): Assume that the input and output feature map dimensions of a standard convolution are (H×W×D×C), where H represents height, W represents width, D represents depth, and C represents the number of channels. Let the subscripts “in” and “out” denote the input and output, respectively. The dimensions of the convolution kernel are (H, W, D, C), where H is the height, W is the width, D is the depth, and C is the number of channels. Let P be the padding value and S be the stride value. The formulas for the output feature maps and the dimensional operations of FLOPs for standard convolution are shown in Equations 1–4:

(1) $$\begin{array}{c} H_{out}=\frac{H_{in}+2\times P-K_{1}}{S}+1 \end{array}$$

(2) $$\begin{array}{c} W_{out}=\frac{W_{in}+2\times P-K_{2}}{S}+1 \end{array}$$

(3) $$\begin{array}{c} D_{out}=\frac{D_{in}+2\times P-K_{3}}{S}+1 \end{array}$$

(4) $$\begin{array}{c} FLOPs\_trad=K_{1}\times K_{2}\times K_{3}\times C_{in}\times C_{out}\times H_{out}\times W_{out}\times D_{out} \end{array}$$

Here, FLOPs_trad refers to the FLOPs of a standard convolution. As shown in **Figure 7b**, efficient feature extraction is achieved through a two-stage process while maintaining the same input and output feature map dimensions and channel counts as in traditional convolutions. A module Deep Convolution (DC): Using a channel grouping strategy, the input feature map is divided into several independent groups along the channel dimension. Each group performs 3×3×3 deep convolution followed by spatial dimension concatenation. While maintaining the same input and output channel counts, parallel processing enhances computational flexibility. Module B: Pointwise Group Convolution (PGC): Channel reorganization is performed based on the greatest common divisor $gcd(C_{in},C_{out})$ of $C_{in}$ and $C_{out}$, and a 1×1×1 convolution kernel is used to achieve cross-channel information fusion and feature dimension mapping. Equation 5 calculates the FLOPs of the DSGC convolution. This design reduces the computational complexity from $\text{O}(C_{in}\cdot C_{out}\cdot K^{3})$ to $\text{O}(C_{in}\cdot (K^{3}+C_{out}))$ while maintaining feature expressiveness (where K is the kernel size).

(5) $$\begin{array}{c} FLOPs\_DSGC=K_{1}\times K_{2}\times K_{3}\times C_{in}\times H_{out}\times W_{out}\times D_{out}+H_{out}\times W_{out}\times D_{out}\times 1\times 1\times C_{out}\times \frac{C_{in}}{gcd(C_{in},C_{out})} \end{array}$$

#### 2.3.4 MHSA

**Figure 8:**
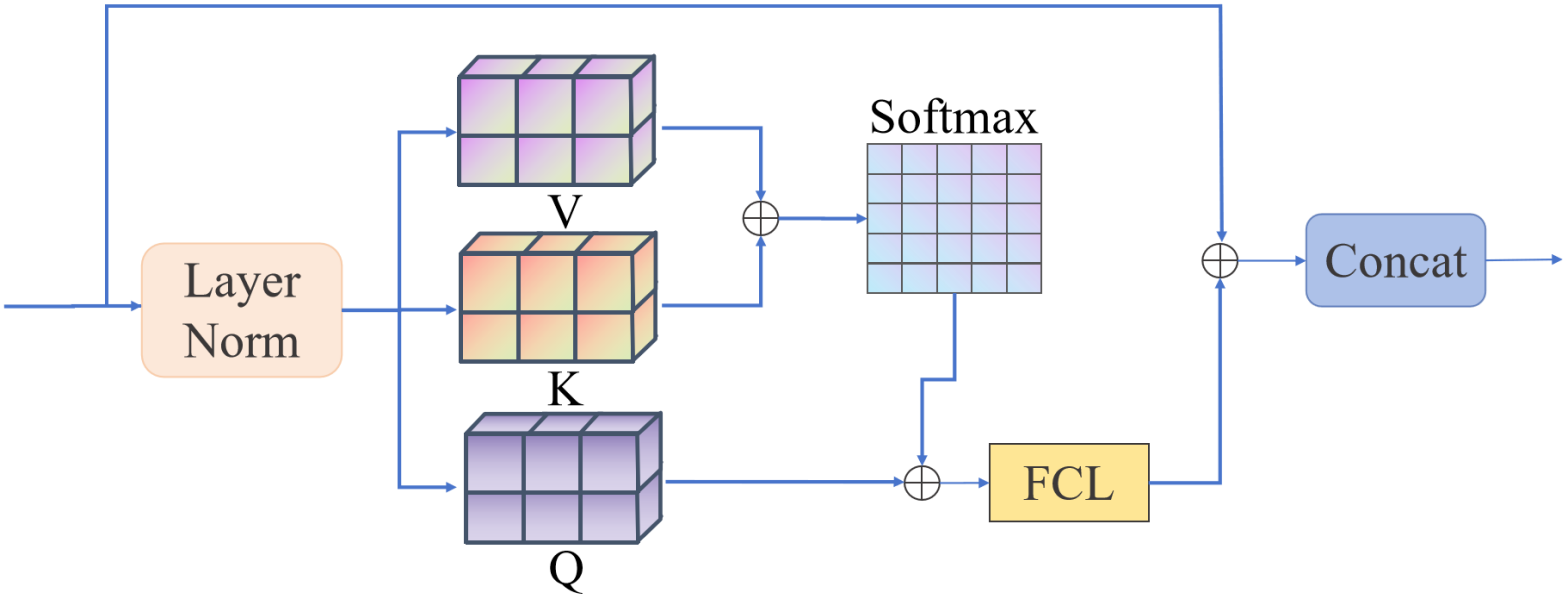
MHSA imaging omics features.

Unlike the self-attention mechanism in traditional Transformer models, the 3D C-Vit network architecture employs a multi-head self-attention mechanism, which captures information from different subspaces to enhance the model’s ability to extract complex features. By mapping input features to multiple heads for parallel processing, each head focuses on a specific feature subspace, thereby achieving a more comprehensive feature representation. Additionally, the MHSA mechanism introduces residual connections and normalization operations during computation and fuses global features through a fully connected layer (FCL). The working principle of MHSA is illustrated in **Figure 8**.

#### 2.3.5 Hyperparameter settings

Through experimental observation, it was found that the model reached a stable performance plateau around 100 training iterations, so the model training iterations were set to 100. Each time the model weights are updated, 16 samples are used to calculate the gradient and perform optimization. To better align with experimental requirements, the BCEWithLogitsLoss loss function, suitable for binary classification, is used to calculate the loss. Since the results from the initial training phase have limited reference value, the cosine annealing method is employed to calculate the learning rate and adapt to the training process, with the default learning rate set to 1e-4. The values output by the sigmoid function for each classification region are interpreted as probabilities.

### 2.4 Ablation experiment

To authenticate the effectiveness of the CAEFF, MSFE, and MHSA modules, we designed systematic ablation experiments. All models adopt the identical training-validation-test data splitting policies for parameter tuning and performance assessments. We constructed the following comparative model schemas, with the outcomes presented in **Table 2**.

**Table 2:** MHSA, MSFE and CAEFF effect on grading of brain tumors in children.

Base	MHSA	MSFE	CAEFF	AUC (%)	Accuracy (%)	Precision (%)	F1 (%)	Recall (%)
✓	×	×	×	80.23	71.69	76.56	78.77	85.49
✓	✓	×	×	81.89	73.33	76.64	75.00	95.74
✓	✓	×	✓	80.22	75.83	76.89	79.34	81.97
✓	✓	✓	×	84.57	80.58	85.07	95.45	98.12
✓	✓	✓	✓	91.36	87.50	95.45	89.29	92.59

Base model (Base): Does not contain the CAEFF, MSFE, and MHSA modules (the specific structure of the base model needs to be clearly defined, such as using only standard 3D convolutional layers).
Base + MHSA: Onboards the MHSA module based on the base model.
Base + MHSA + MSFE: Onboards the MSFE module on the basis of having onboarded the MHSA module.
Base + MHSA + CAEFF: Onboards the CAEFF module on the basis of having onboarded the MHSA module.

Complete model (Ours): Base + MHSA + MSFE + CAEFF (i.e., 3D C-Vit model).By comparing the AUC and accuracy metrics of each mock-up on the test set (**Table 2**), the contribution of each module to the final performance is quantized.

### 2.5 Comparison model construction

#### 2.5.1 Clinical model construction

The clinical model selected seven features: age, gender, type, location, necrosis, edema, and Ki-67. Univariate analysis was performed using the chi-square test to identify statistically significant features (*p* < 0.05), and a clinical model was then constructed using RandomForest (24).

#### 2.5.2 Imagingomics comparison model construction

In this study, first-order features, shape-based features, gray-level co-occurrence matrix (GLCM), gray-level run length matrix (GLRLM), and wavelet features were extracted from regions of interest in five sequences. First, a Chi-square test was used for univariate analysis, followed by binary least absolute shrinkage and selection operator (LASSO) regression for multivariate analysis. CLS token features were extracted from the optimal model, and after feature standardization, five-fold cross-validation training was performed to select important features. Imaging models were established to distinguish between low-grade and high-grade pediatric brain tumors using RandomForest (24), SVM (25) and DecisionTree (26).

#### 2.5.3 Construction of a deep learning-based comparison model

The dataset allocation and hyperparameter settings were synchronized with the 3D C-Vit model. Deep learning models—including Modified VGG16 (27), DenseNet121 (28), ResNet50 (2), Transformer-based (29) and Swin Transformer (30)—were developed to distinguish between low-grade and high-grade pediatric brain tumors.

### 2.6 Model evaluation

To assess the diagnostic capability of the models, the area under the receiver operating characteristic curve (AUC) and confusion matrix were calculated. To evaluate model performance, quantitative metrics such as classification accuracy, precision, F1 score, and recall rate were calculated for all models. The variable equations are shown in formulas Equations 6–10.

(6) $$AUC=\frac{\sum_{i\in SPS}rank_{i}-\frac{M*(M+1)}{2}}{M*N}$$

(7) $$Accuracy=\frac{\left(TP+TN\right)}{\left(TP+TN+FP+FN\right)}$$

(8) $$Recall=\frac{TP}{\left(TP+FN\right)}$$

(9) $$Precision=\frac{TP}{\left(TP+FP\right)}$$

(10) $$F1Score=2\times \frac{\left(Precision\times Recall\right)}{\left(Precision+Recall\right)}$$

Among them:

- SPS represents the set of positive samples, M represents the number of positive samples, N represents the number of negative samples, and rank represents the sample with the highest probability in the prediction.
- True positive (TP) is a case where the prediction is correctly positive.
- True negative (TN) is a case where the prediction is correctly negative.
- False positive (FP) is a case where the prediction is incorrectly positive.
- False negative (FN) is a case where the prediction is incorrectly negative.

### 2.7 Statistical analysis

In this study, the Chi-square test was used to analyze the distribution differences of categorical variables among the training set, validation set, and test set; the independent samples t-test was used to analyze the differences in continuous variables (age) between groups. The LASSO regression algorithm was used for feature dimension reduction, and the optimal regularization coefficient λ was determined through five-fold cross-validation to screen out features with non-zero coefficients. The construction of the radiomics prediction model was based on three mainstream machine learning frameworks, including RandomForest, SVM, and DecisionTree. The diagnostic performance of the model was evaluated by quantitative indicators such as AUC, accuracy, precision, F1-score, and recall. All feature variables included in the final diagnostic model were strictly screened by statistics, and their significance thresholds were all controlled at the level of p < 0.05.

Interpretability was obtained through radiomics models and 3D C-Vit model, respectively. The weight coefficients of the 59 important features selected by LASSO regression and their distribution across sequences (ADC, T2, etc.) were analyzed. Based on the SE attention weights in CAEFF, the GN modulation weights in MSFE, and the attention maps of MHSA, interpretability analysis was performed to locate the key tumor regions and features focused on by the model decision-making.

## 3 Results

### 3.1 Patient characteristics

As shown in **Table 1**, a total of 340 cases were included in this study, with 237 cases for training, 51 cases for the validation set, and 52 cases for the test set. Male children accounted for 62.9% of the total data, which was 214 cases, and the remaining were female patients, aged between seven months and 16 years. Tumors of grade I/II were classified as low-grade brain tumors, and grade III/IV as high-grade tumors, with low-grade pediatric brain tumor accounting for 42.1% of the total data. There were three locations of tumors in the brain: cerebellum accounted for 38.5% of the total, fourth cerebral ventricle accounted for 58.2%, and brainstem accounted for 3.4%. The dataset included three tumor types: EP accounted for 21.2%, MB accounted for 46.8%, and PA accounted for 32%. The three cohorts were evenly distributed in terms of gender, age, tumor grade, tumor location, and tumor type (see **Table 1** for details).Chi-square test and t-test results showed that the differences in characteristics between the cohorts were statistically significant in tumor location and tumor type (p < 0.05).

### 3.2 Comparison of existing models

To comprehensively evaluate the performance of the proposed 3D C-Vit model, we compared its performance with the clinical model, radiomics models, and various deep learning comparison models on the same test set. The results are summarized in **Table 3**.

**Table 3:** Performance comparison of different models for pediatric brain tumor (LG vs. HG) grading on the test set.

Model type	Method	AUC (%)	Accuracy (%)	Precision (%)	F1 score (%)	Recall (%)
Clinical	Univariate analysis + RandomForest	81.04	73.23	74.58	73	79
Radiomics	RandomForest	82.35	76.92	82.14	76.67	79.31
	SVM	86.14	77.55	85.19	80.7	76.67
	DecisionTree	72.1	69.39	74.19	75.41	76.67
DL	Modified VGG16	88.69	76.51	76.67	80.70	84.99
	DenseNet121	84.42	77.53	80.78	80.3	80.01
	ResNet50	76.9	75	90	80	**96.30**
	Transformer-based	86.15	82.61	87.5	86.21	72.88
	Swin Transformer	89.79	83.54	80.78	76.92	75.09
**Ours**	**3D C-Vit**	**91.36**	**86.53**	**95.45**	**89.29**	92.59

*DL indicating deep learning. Bold text indicates the best values across all models.

As shown in **Table 3**, the 3D C-Vit model achieved the best or near-best results in all evaluation metrics. Its AUC (91.36%) was significantly higher than that of all other models, indicating its strongest overall discriminative ability to distinguish between LG and HG. Meanwhile, it achieved the highest accuracy (86.53%), precision (95.45%), and F1-score (89.29%), with the recall (92.59%) also at a high level. This indicates that the 3D C-Vit model performs excellently in reducing Incorrect diagnosis and missed diagnosis, achieving a good balance.

This model achieved a classification accuracy of 86.53% on the test set, with approximately 7 out of 52 test samples being misclassified. This is primarily attributed to factors such as the similarity of visual features, the complexity of the image data, the lack of quantitative feature analysis, differences in imaging equipment, and individual patient variations. Among the misclassified samples, PA had the highest probability of misclassification, at approximately 25%. This was primarily because its low-grade tumors exhibited the atypical characteristics of high-grade tumors, with imaging features such as irregular borders or increased volume in low-grade tumors. The misclassification rate for EP was approximately 17%, with most misclassified samples being small, high-grade tumors with clear borders; The misclassification rate for MB is relatively low, at approximately 10%, with misclassified cases typically being low-grade tumors characterized by blurred borders and a lack of hallmark features of malignant growth. Additionally, when quantifying using the border gradient coefficient, the average value for misclassified low-grade tumors was 0.42, while that for misclassified high-grade tumors was 0.18. High-grade tumors in young patients under 10 years of age are prone to being misclassified as low-grade, which may be related to atypical tumor growth patterns and imaging features. In summary, these factors may obscure the model’s ability to accurately distinguish tumor grades, leading to misclassification. This analysis highlights the challenges faced in distinguishing tumors with subtle visual differences and suggests directions for future model improvements.

Through comparative studies of three types of models, it was found that the clinical model had the weakest performance, with model performance (AUC 81.04%) significantly lower than that of models based on imaging data, highlighting the value of multi-sequence MRI imaging information in tumor grading. Among the radiomics models, the SVM model (AUC 86.14%) performed the best, but its overall performance was still significantly inferior to 3D C-Vit. This indicates that although manually designed radiomic features have certain value, their representational ability is limited, making it difficult to fully capture the complex 3D spatial heterogeneity and deep texture patterns of tumors. The Swin Transformer, which is based on a deep learning model, performed relatively well, achieving an AUC of 89.79% and an accuracy of 83.54%, second only to 3D C-Vit, though it lagged significantly behind in other metrics. Our model achieved an F1 score of 89.29%, approximately four percentage points higher than the second-ranked Transformer-based model (86.21%); its precision of 95.45% was significantly higher than that of other models. ResNet50 (AUC 76.90%) had the highest recall rate (96.30%), meaning it could identify the vast majority of brain tumor cases, but its precision (90.00%) and accuracy (75.00%) were low, indicating a high false positive (FP) rate, which easily misjudged some LG as HG, which may lead to over-treatment in clinical applications.DenseNet121 (AUC 84.42%) has moderate performance.

**Figure 9:**
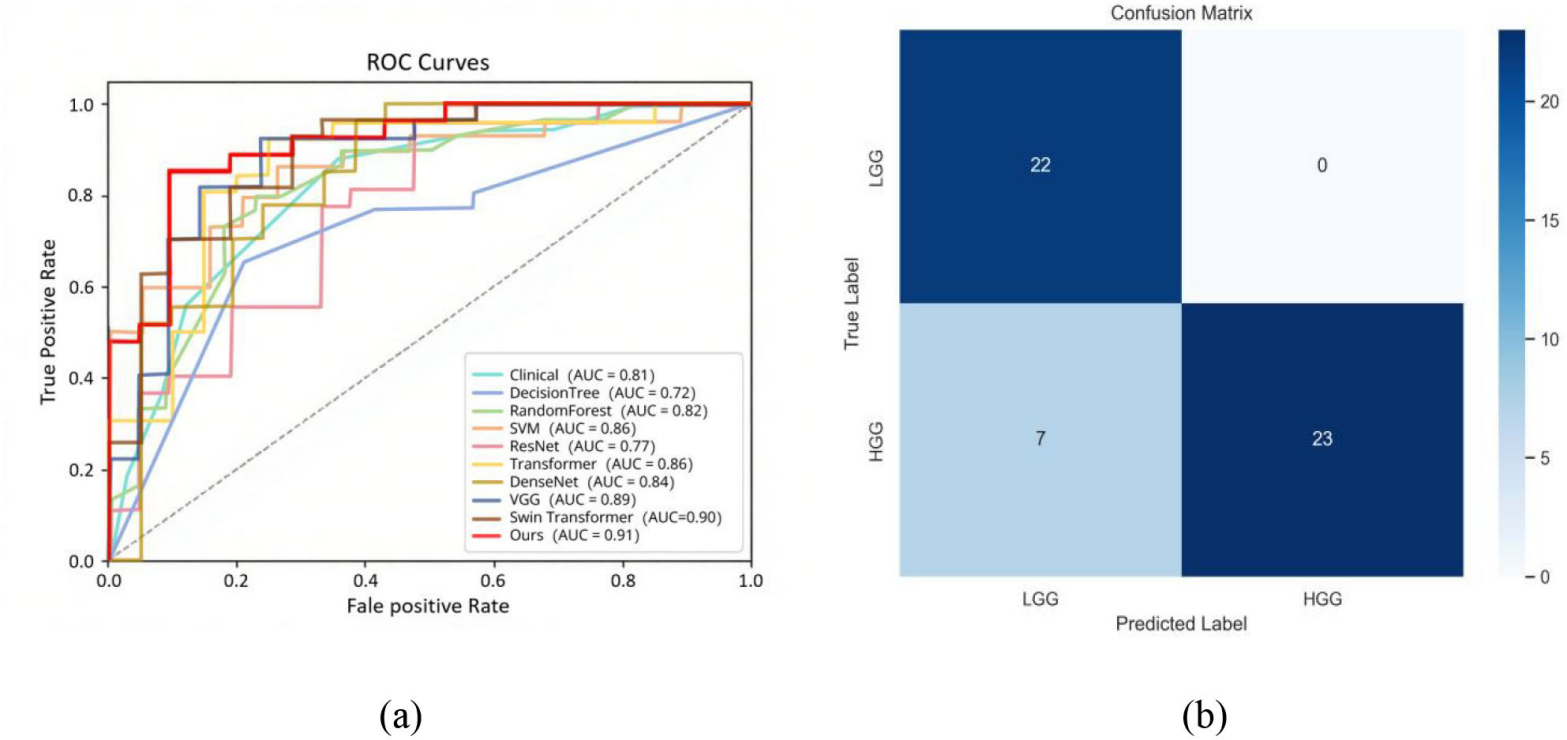
**(a)** ROC curves of all models. Red represents 3D C-Vit model, the corresponding curves and AUC values of other comparative models are shown in the lower right corner of the graph. **(b)** Confusion matrix of 3D C-Vit model test. The abscissa represents the predicted label, the ordinate represents the true label. With darker colors indicating higher values.

**Figure 9a** shows the comparison of ROC curves of different models for pediatric brain tumor grading on the test set. **Figure 9b** Confusion matrix of the prediction results of the 3D C-Vit model on the test set. The curve of the 3D C-Vit model (red) is closest to the upper left corner, further confirming its superior discriminative ability. The curves of other models are relatively deviated, especially the areas under the curves of the ResNet50 and Decision Tree models are significantly smaller.

To comprehensively evaluate the efficiency of the 3D C-Vit model, we compared it with classical CNNs and mainstream Transformer-based models across two key metrics: the number of parameters (Params) and computational complexity (FLOPs). As shown in **Table 4**, the 3D C-Vit model has 1.82 million parameters and a computational complexity of 28.5G FLOPs, both of which are lower than those of other models; Although the computational complexity of Transformer-based models (33.62G FLOPs) is close to that of the 3D C-Vit model, their number of parameters is significantly higher than that of the 3D C-Vit model. These results indicate that our model effectively optimizes the computational workflow while reducing the number of parameters, thereby significantly lowering computational complexity.

**Table 4:** Comparison of Model Complexity.

Model	Modified vgg16	Densenet121	Resnet50	Transformer-based	Swin transformer	3D c-bit
Params (M)	61.82	69.73	30.08	12.65	29.48	1.82
FLOPs (G)	420.74	151.25	58.94	33.62	231.49	28.5

### 3.3 Model evaluation

**Figure 10:**
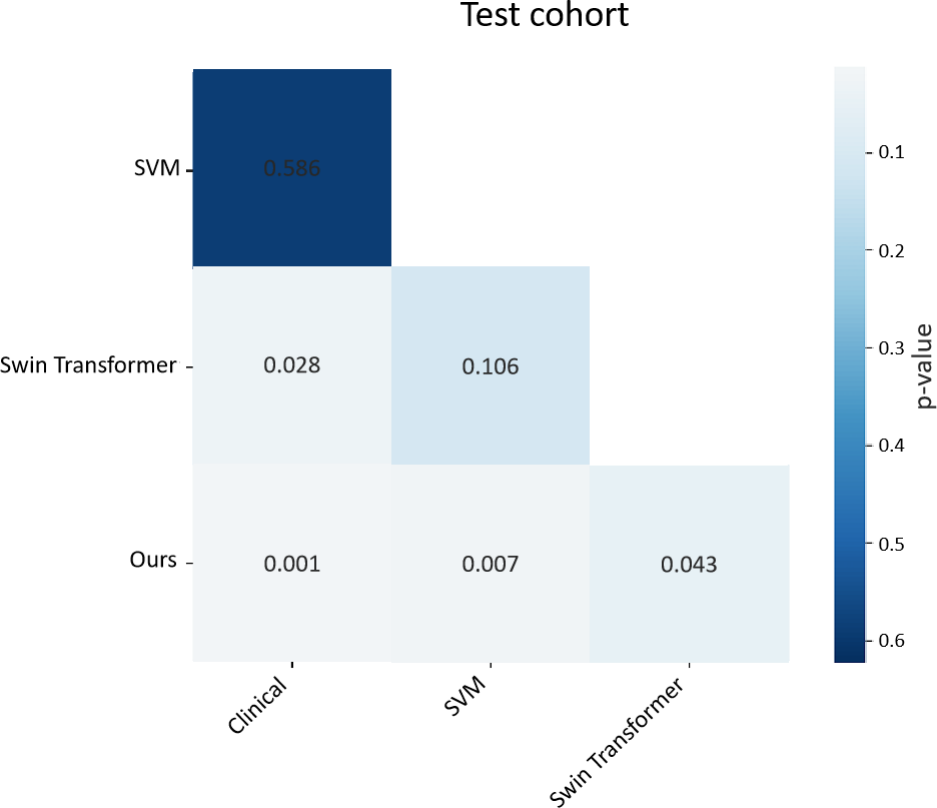
Delong result.

To evaluate the diagnostic performance of the Clinical, Radiomics, deep learning, and Ours models, we selected the Clinical model, the optimal radiomics model (SVM), the optimal deep learning model (Swin Transformer), and the 3D C-Vit model for DeLong’s test to compare the discriminatory capabilities of their ROC curves. As shown in **Figure 10**, the predictive performance of the Swin Transformer model (p = 0.028) was significantly better than that of the Clinical model. The p-values for the SVM model, the Clinical model, and the Swin Transformer model were 0.586 and 0.106, respectively; there was no statistically significant difference in predictive performance among them (p > 0.05). The p-values for the 3D C-Vit model compared to the Clinical, SVM, and Swin Transformer models were all less than 0.05, demonstrating that our model’s predictive performance is significantly superior to that of the other models.

### 3.4 Ablation experiment

To verify the effectiveness of the proposed CAEFF, MSFE, and MHSA modules and their contribution to model performance, we conducted systematic ablation experiments, with the outcome displayed in **Table 2**. Adding the MHSA module alone to the base model elevates the AUC to 81.89% (+1.66%) and the accuracy to 73.33% (+1.64%). Noteworthy is that the recall increases significantly from 85.49% to 95.74% (+10.25%) after adding MHSA, indicating that the multi-head self-attention mechanism has advantages in capturing tumor-surrounding organization long-range dependence relationships and global context information, which helps reduce missed diagnoses of HG.

The CAEFF module effectively enhances feature fusion. After adding the CAEFF module, the AUC slightly decreases to 80.22%, but the accuracy increases to 75.83% (+4.14%).This module reinforces the T1WI channel feature through SE attention and dynamically processes other channels via a gate network, effectively fusing the complementary information of multi-sequence MRI to enhance the representation of discriminative features.

The MSFE module makes the greatest contribution and enhances global modeling. After adding the MSFE module, the AUC increased from 80.23% to 84.57% (+4.34%), the accuracy increased from 71.69% to 80.58% (+8.89%), and the F1-score and recall rate were significantly improved (F1 + 16.68%, Recall +12.63%). This fully attests that through its innovative Gated Network (GN) and Depthwise Separable Group Convolution (DSGC) design, the MSFE module plays a key role in efficiently extracting multi-scale local features (such as tiny necrosis and hardening edges), which greatly enhances the model’s ability to perceive the internal heterogeneity of tumor.

The module synergy effect is significant. When all modules (MHSA + MSFE + CAEFF) are added to the base model simultaneously, the performance reaches the optimal level (AUC 91.36%, Accuracy 87.50%), far exceeding the contribution of any single module (the maximum AUC elevation comes from MSFE + 4.34%, and the maximum Accuracy elevation comes from MSFE + 8.89%).This strongly attests to the rationality and synergistic effect of the CAEFF, MSFE, and MHSA module designs. CAEFF provides optimized multi-sequence feature input, MSFE efficiently extracts key local multi-scale features, and MHSA grabs global context dependencies. The combination of the three realizes effective Fusion of local details and global information, significantly elevating the overall banding performance of the mock-up.

### 3.5 Interpretability

#### 3.5.1 Interpretability of the radiomics models

**Figure 11:**
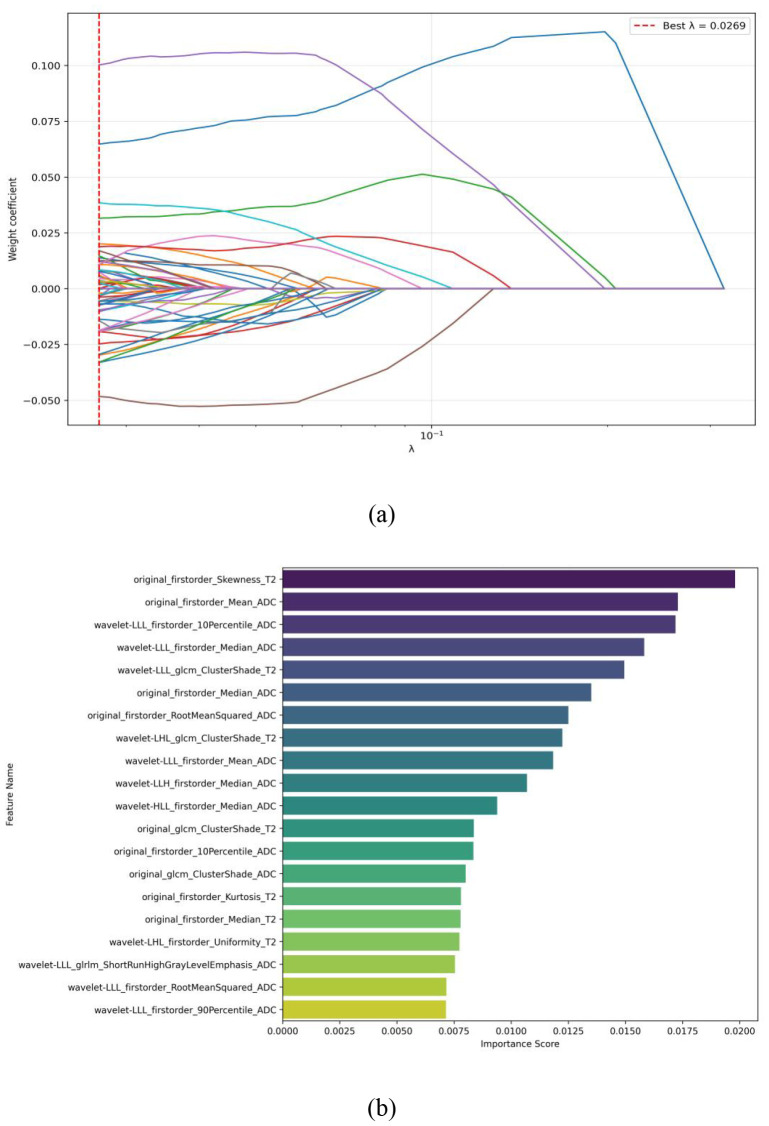
Radiomics features. **(a) **Features selected by the LASSO regression. **(b) **Top 20 most important features ranked by absolute LASSO coefficient values.

After the Chi-square test, two features were considered statistically significant (p < 0.05) (**Table 1**), and the two features with significant differences were adopted by the clinical model. In the radiomics models, a total of 1074 features were extracted for each VOI. After chi squared univariate analysis, 691 features were considered statistically significant (p < 0.05). LASSO regression indicated that 59 features were effective metrics for distinguishing low-level and advanced lesions, as shown in **Figure 11a**. Among them, the top 20 most important features ranked by the absolute values of LASSO coefficients are shown in **Figure 11b**, with ADC sequence features and T2 sequence features occupying significant positioning. The quantity of ADC features is large and their significance is high, and T2 features also account for a certain proportion, reflecting that the model, in the pediatric brain tumor grading task, Focused on grabbing the variation in imaging phenotypes of lesions at the level of “water molecule diffusion sports (ADC sequence) and tissue signal contrast (T2 sequence)”. ADC sequence reflects the diffusion movement of water molecules. LG has relatively low cell density, resulting in mild restriction of water molecule diffusion (relatively high ADC value); HG has high cell density, obvious nuclear atypia, and active cell proliferation, leading to significant restriction of water molecule diffusion (relatively low ADC value). The characteristics of ADC sequence can sensitively reflect the variation in cell density and microstructure.T2WI sequence reflects the tissue signal contrast. LG usually has relatively clear boundary and relatively uniform internal signal; HG often presents complex signal changes such as heterogeneous signal, necrotic cystic degeneration, infiltrative growth, and surrounding edema. The characteristics of T2WI sequence can effectively grab these morphological and signal heterogeneities.

#### 3.5.2 Interpretability of the 3D C-Vit model

Unlike radiomics feature analysis, when extracting features in the 3D C-Vit model CAEFF module, SE compacts the spatial information of the feature map through global average pooling to generate channel-level global descriptions. In the MSFE module, GN captures the global context of the input feature map through global average pooling to generate spatial-channel syndication attention weights, and DSGC extracts complex image features through depth-wise convolution and point-wise convolution. In addition, MHSA extracts information by automatically spotlighting the most discriminative territory in the input image. The experiment outcome shows that the onboarding of each module can elevate the performance of the model (**Table 2**).

The SE attention mechanism enhances the model’s sensitivity to key structures (such as enhancement rings, internal heterogeneous regions of tumor) in T1WI sequences by learning channel weights. Channels with higher SE weights usually correspond to regions where tumor enhancement is most significant or internal structures are most complex. For the MSFE module, the spatial-channel joint attention weights dynamically generated by the gating network enable the model to adaptively focus on discriminative local regions within tumor (such as tiny necrosis foci, invasive fronts) while suppressing background or irrelevant regions. Depthwise separable grouped convolution (DSGC) efficiently extracts texture and edge details of these local regions. The MHSA module, based on the multi-head self-attention mechanism, reveals the long-range dependence relationships within tumor and between tumor and surrounding tissues that the model focuses on by calculating attention weights in different subspaces. The model tends to focus on the boundary area of tumor, the junction between internal necrosis and enhanced regions, as well as the interface interacting with surrounding oedema or compressed tissues. These regions usually contain abundant pathological information and are important clues for distinguishing tumor grades.

**Figure 12:**
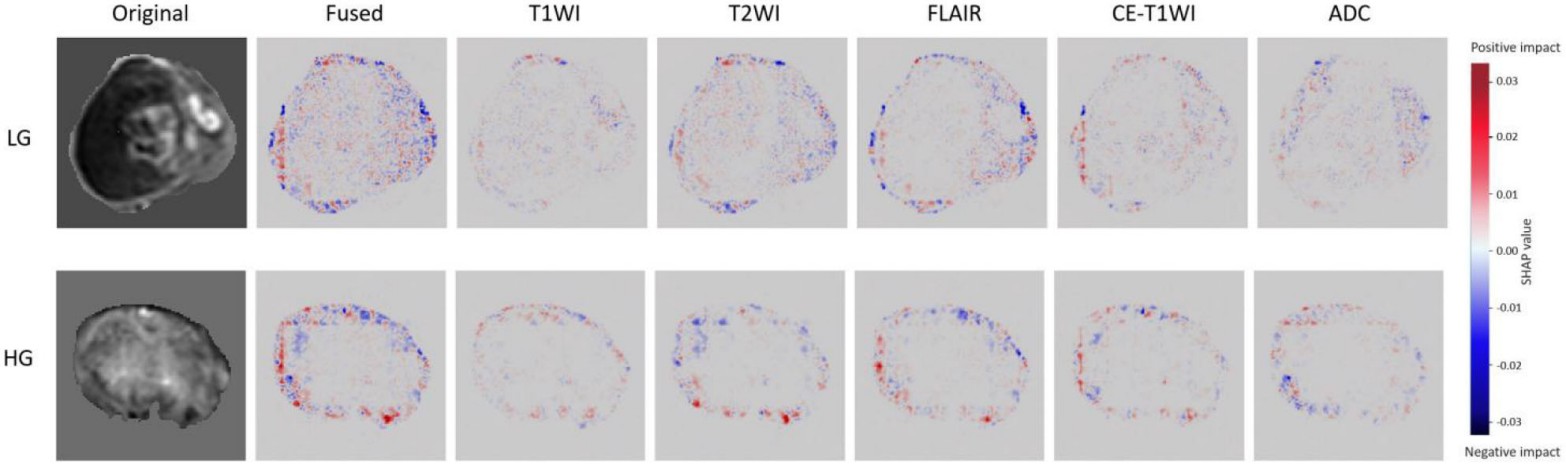
SHAP analysis.

To quantify the relationship between multimodal features and model decisions, this study employs the SHapley Additive Explanation (SHAP) method to perform a visual attribution analysis of model predictions, thereby quantifying the contribution of different modalities and spatial regions to the final prediction results. The analysis selected the slice containing the most diagnostic information for visualization, presenting the original MRI image, a fused SHAP heatmap, and independent SHAP heatmaps for the five modalities and three image categories (31). In these visualizations, red regions represent a positive contribution to the prediction of the current category, while blue regions represent a negative contribution; the intensity of the color is proportional to the strength of the contribution. As shown in **Figure 12**, the high-contribution red and blue regions in the SHAP heatmap are highly concentrated at the interface between the tumor margin and the surrounding brain tissue, intuitively demonstrating that the model focuses primarily on tumor boundary features. Further quantification of the contribution of each modality shows that T1WI accounts for 15%, T2WI for 22%, FLAIR for 25%, CE-T1WI for 20%, and ADC for 18%. In terms of feature distribution, the model focuses on lesion areas with clear clinical significance, such as enhancement foci in CE-T1WI, edema bands in T2WI/FLAIR, and cystic areas in T2WI. Its decision-making logic aligns closely with the clinical diagnostic judgment of radiologists, which not only validates the model’s reliability and interpretability but also provides a quantitative reference for optimizing MRI sequence selection in preoperative clinical examinations.

The outcomes of the ablation experiment (**Table 2**) indirectly confirm the effectiveness of each module and their contribution to model performance, and indirectly support the role of these modules in grabbing different levels of feature (local details, channel information, global context). In the future, more sophisticated visualization techniques (such as Grad-CAM++, 3D Attention Rollout) can be used to further quantize and show the specific 3D spatial positioning that the mock-up focuses on.

## 4 Discussion

Early and accurate staging plays a decisive role in developing personalized treatment plans, assessing prognosis, and improving survival rates for pediatric brain tumors. However, traditional imaging diagnosis relies heavily on manual interpretation, and different radiologists’ interpretations of tumor morphological features are easily influenced by experience thresholds, environmental factors, and cognitive load, leading to significant variations in diagnostic outcomes (32, 33). Furthermore, the high heterogeneity of tumors and the complexity of the brain tissue background make it difficult for traditional classification methods based on visual features to handle borderline cases. Manual processing of massive amounts of imaging data is not only inefficient and time-consuming but also lacks reproducibility, increasing the risk of diagnostic delays. These issues not only threaten diagnostic accuracy but may also lead to biases in treatment decisions. Studies have shown (34) that workflows reliant on subjective experience can no longer meet the demand for standardized, quantifiable diagnostic indicators in the era of precision medicine; therefore, there is an urgent need to introduce intelligent, objective auxiliary diagnostic systems.

To address such issues, machine learning has been widely applied in brain tumor research (35–37). Convolution operations were introduced due to performance limitations imposed by various factors (5, 38–42), For example, Dong et al. (43) proposed a deep learning framework based on ResNet that achieves detection and classification of pediatric brain tumors using 2.5D MRI data.Ali et al. (44) proposed a multi-stage framework based on a 26-layer CNN that leverages transfer learning to extract features from MRI images for Alzheimer’s disease detection and classification. Methodologically, this approach is similar to our proposed 3D C-Vit model. This comparison not only demonstrates the effectiveness of multi-stage networks in handling complex image features but also offers insights for further optimizing brain tumor diagnosis models. For instance, integrating multi-stage feature extraction strategies with attention mechanisms could better model intratumoral heterogeneity and inter-patient variability, thereby improving diagnostic accuracy and model generalizability. However, the local receptive field mechanism inherent in CNNs restricts convolutional kernels to capturing only spatial features, making it difficult to model semantic correlations across channels. Insufficient utilization of channel information leads to performance bottlenecks when handling tumor heterogeneity and low-contrast images. However, traditional CNNs have limitations: the local receptive field of convolutional kernels restricts their ability to model cross-channel semantics, and the underutilization of channel information reduces their efficiency when handling tumor heterogeneity and low-contrast regions; furthermore, the inductive bias of convolutional operations limits the modeling of global semantic information (45). Research has shown (46) that to construct image representations with generalization capabilities, it is necessary to break away from the “local-first” design paradigm. Consequently, Transformer architectures based on global information have emerged (47–49). However, the Transformer’s insufficient accuracy in local information extraction may affect the recognition of small lesions (50). Although Tonmoy et al. (51) attempted to fuse CNNs with Transformers, their approach still relied on convolutional operations and failed to fully leverage the potential of the attention mechanism. Azam et al. (52) proposed a method combining Transformers and CNNs that demonstrated effectiveness and improvements in the grading of pediatric brain tumors; however, its ability to extract local information remains limited.

The 3D C-Vit model proposed in this paper significantly enhances the capture of local details by introducing the CAEFF channel attention-enhanced feature fusion module and the MSFE multiscale feature extraction module. The MSFE module employs dynamic convolution to improve computational efficiency while strengthening the interaction between global contextual information and local features, achieving tight fusion through dynamic weight allocation. This method effectively addresses the shortcomings of traditional convolutions in global semantic modeling and resolves the coarse handling of local details in pure Transformer models. Experimental results demonstrate that the improved model exhibits significantly enhanced sensitivity in detecting small lesion regions, providing a reliable basis for the early diagnosis of pediatric brain tumors. Compared with existing state-of-the-art 2D and 3D methods, 3D C-Vit outperforms other models across all comprehensive metrics. Regarding model interpretability, radiomics models rely on manually selected features, with their interpretability primarily reflected in the quantification of specific imaging metrics. In contrast, the 3D C-Vit model automatically captures both global and local key information through self-attention mechanisms and CNNs, demonstrating greater adaptability and robustness.

This study has the following limitations. Grade was determined solely based on MRI data to distinguish low-grade (LG) tumors (including Grade I and II) from high-grade (HG) tumors (including Grade III and IV). This classification approach enables the model to identify key features in the imaging data, thereby helping clinicians more effectively assess tumor grade and develop personalized treatment plans based on different grades. In the future, as more cases are collected and studied, further molecular subtyping based on genes and proteins can be conducted to enable more precise staging and personalized treatment. A third limitation is that all data were collected from a single institution; the model’s generalizability to external environments still requires further evaluation. Future work will involve validating the model using multi-institution, multi-scanner datasets to enhance its robustness and clinical applicability.

Overall, the 3D C-Vit model proposed in this study, which combines Transformers and CNNs, offers a new approach for the accurate diagnosis of pediatric brain tumors. Although this study is limited by a small sample size, which may affect the model’s reliability, we mitigated the risk of overfitting by employing techniques such as Dropout, L2 regularization, and Cosine Annealing. Furthermore, we repeated the experiments 20 times and took the average to assess the stability of the results, thereby validating the reliability of the method. Importantly, we successfully combined the global perspective of Transformers with the local perspective of CNNs and validated the heterogeneity of its decision-making mechanisms and clinical cognition through interpretability analysis. Future research will focus on expanding the sample size and collecting more multicenter data from different regions and medical institutions to enhance the robustness and generalizability of the results. With the accumulation of data and the advancement of multicenter collaboration, we anticipate further validating and optimizing this model, as well as evaluating its applicability in larger populations.

## 5 Conclusions

This paper proposes a hybrid network architecture—3D C-Vit —that combines Transformers and CNNs for the efficient grading of pediatric brain tumors, capable of capturing both global and local features simultaneously. The model achieves efficient differentiation between low-grade and high-grade lesions through three key modules: MSFE, CAEFF, and MHSA, and validates the performance improvements of these modules through ablation experiments. Additionally, to ensure fairness in evaluation, the most commonly used binary classification methods for pediatric brain tumors were applied to the same dataset, and their performance was evaluated using a unified metric.

The MSFE module employs efficient dynamic convolution to enhance the efficiency of feature calculation and grading (**Table 2**). The CAEFF module utilizes the SE attention mechanism to balance the variability across different sequences. The highest accuracy was achieved when all modules were used (**Table 2**), with an accuracy rate of 86.53% in the grading of pediatric brain tumors. Exploratory analysis reveals that 3D C-Vit differs significantly from traditional radiomics in terms of feature extraction and decision-making mechanisms, highlighting the advantages of deep learning in complex medical image analysis. Compared to traditional diagnostic workflows that rely on manual interpretation—which are time-consuming and highly subjective—3D C-Vit provides quantitative, objective decision support, improving diagnostic timeliness and accuracy, and offering a new paradigm for clinical practice and medical imaging research.

## Data Availability

The data analyzed in this study is subject to the following licenses/restrictions: The datasets presented in this article are not readily available because The datasets generated and/or analyzed during the current study are not publicly available due to the ongoing further studies but are available from the corresponding author on reasonable request. Requests to access these datasets should be directed to Jie Dong, dongjie@ncwu.edu.cn.
