# Supplementary material for: Interpretability and performance of a 3D C-vit model for accurate grading of pediatric brain tumors
Source: Front Oncol. 2026 Apr 27;16:1763280. doi: 10.3389/fonc.2026.1763280 (PMC13158093; doi:10.3389/fonc.2026.1763280)
Supplement: Supplementary file 1 [file DataSheet1.docx]

Supplementary Material

# Supplementary Data

1.Six Different Types of MRI Scanners: 3.0 T Discovery MR 750 (GE Healthcare), 3.0 T Skyra (Siemens Healthcare Solutions), 3.0 T Trio TIM (Siemens Healthcare Solutions), 3.0 T Prisma (Siemens Healthcare Solutions), 3.0 T Verio (Siemens Healthcare Solutions), and 3.0 T Ingenia (Philips Healthcare system). The field of view of all scanners is 220-240mm.

2.Setting parameters for image intensity normalization: Wstripe_Radius = 0.05, Wstripe_tissuesMax = 5, Wstripe_smoothMax = 10, Wstripe_smoothDelta = 0.5, Wstripe_hietSize = 2000, and both Wstripe_sliceStartZ and Wstripe_sliceStopZ were set to -1.
